# Comparison and Evaluation of Current Animal Models for Perineural Scar Formation in Rat

**Published:** 2013-07

**Authors:** Leila O Zanjani, Masoumeh Firouzi, Mohammad-Hossein Nabian, Mohsen Nategh, Vafa Rahimi-Movaghar, Reza S Kamrani

**Affiliations:** 1 Tissue Repair Lab, Institute of Biochemistry and Biophysics, University of Tehran, Tehran Iran; 2 Research Center for Neural Repair, University of Tehran, Tehran, Iran; 3 Department of Orthopedics and Trauma Surgery, Shariati Hospital, Tehran University of Medical Sciences, Tehran, Iran; 4 Sina Trauma and Surgery Research Center, Tehran University of Medical Sciences, Tehran, Iran

**Keywords:** Scar, Sciatic nerve, Translational research

## Abstract

***Objective (s): ***Scar formation in injured peripheral nerve bed causes several consequences which impede the process of nerve regeneration. Several animal models are used for scar induction in preclinical studies which target prevention and/or suppression of perineural scar. This study evaluates the translational capacity of four of physical injury models to induce scar formation around the sciatic nerve of rat: laceration, crush, mince and burn.

***Materials and Methods:*** Functional (Toe out angle), macroscopic, and microscopic evaluations were performed weekly for four weeks and correlation of findings were analyzed.

***Result:*** While macroscopic and microscopic findings suggested a well-developed and adhesive fibrosis surrounding the sciatic nerve, functional assessment did not reveal any significant difference between control and experimental groups (*P*>0.05).

***Conclusion: ***Our study suggests that none of the applied animal models reproduce all essential features of clinical perineural scar formation. Therefore, more studies are needed to develop optimal animal models for translating preclinical investigations.

## Introduction

Peripheral nerve repair has always been a challenging problem in the context of scar formation at the injury site. Clinical consequences of scar formation include chronic compression, preventing mobility and thus tethering of nerves due to adhesion to adjacent tissues (1). In the case of peripheral nerve injuries successful interventions and strategies should lead to improvement of quality of life in patients with chronic nerve injuries (2, 3). Animal models are the primary preclinical pathway in translating regenerative medicine to reduce scar accompanied in nerve injuries. Thus, effective and reliable translational animal models should meet two criteria: First, they should provide an environment that matches, to the greatest extent possible, the clinical and biomechanical properties in which interventions are assessed in the nerve bed. Second, they should provide objectives and parameters to assess functional performance resulting from interventions (4).

Currently preclinical research of nerve regeneration is generally performed on the sciatic nerve of rat. To mimic scar formation scientists have applied a variety of interventions. Interventions have been either chemical such as administration of talc powder (5), tetracycline (6) and silver nitrate(7); or physical, as in crushing (8), abrading (9-11), lacerating (12) and burning (1, 8, 9) of muscles which are more comparable to clinical setting. Yet, little information has been published on the nature of such scar-inducing interventions concerning their real impact on nerve function and its correlation with morphologic properties of scar. More importantly, the translational capacity of these models should be questioned when we compare great regeneration potential of rodents with humans. In this preliminary study, using a behavioral assessment, we examined whether lacerating, crushing, mincing and burning of muscles, as physical interventions for scar induction around the nerve, affect sciatic nerve function in rat and thus mimic the key features of clinical settings of scar formation.

## Materials and Methods

A total number of thirty two female Wistar rats weighing 150-200 g were randomly divided into four groups (n=8) each sustaining any of laceration, crush, mince and burn injuries. Animals were handled according to the laboratory animal care and treatment protocol of Tehran University of Medical Sciences. After anesthesia (Ketamine100 mg/kg, and Xylazine 5 mg/kg, IP), and under aseptic conditions, the right sciatic nerve was carefully exposed and isolated through a gluteal muscle-splitting approach. Over a length of 1.5 cm of the exposed surface of the adductor muscle and 5 mm edge of the biceps femoris muscle, both facing the sciatic nerve, four interventions were applied. For the laceration group, surgical blade (#15) was used to make five axial and three vertical slashes, each 2 mm-deep in both muscles (12). For the crushing group, the two muscles were compressed with forceps at its maximum closure point for 10 sec (8). For the mincing group, 5 mm edge of the biceps femoris and whole adductor muscle were severed and taken out, then minced into small pieces and returned back to the nerve periphery. As a modified burning procedure, a wide-tip pincer (5×5 mm) was heated by flame until became reddish and pressed onto the muscles (8, 9). During these procedures the nerve was gently retracted and protected against direct injury. Muscles and skin were sutured and an anti-bite (Mavala Stop Inc UK) was administered on the operated limb for prevention of probable automutilation.

For four weeks, behavioral, histological and macroscopic assessments were performed weekly on animals: the Toe Out Angle (TOA) test was engaged as the behavioral assessment. By definition, “The angle in degrees between body’s direction of progression and the line which passes through the tip of third digit and the calcaneus is defined as TOA” (13). For monitoring of this angle a Perspex walking pathway was placed in front of a recording camera (SONY, DCR-DVD305, Japan, Tokyo). A mirror with 45 degree angle was placed beneath the walking track which provides the bottom view of animals’ feet plantar surface for the recorder. Continuous stepping of animals was recorded and frames in which the animal was running, crouching or intermittently stepping were considered inappropriate and removed from calculations. The TOAs were then calculated using the public domain software ImageJ (www.rsb.info.nih.gov accessed Nov 2007). TOA measurement of another nineteen rats served as control in our study.

 At the end of each week, two animals from each group were sacrificed; one for macroscopic and the other for microscopic evaluations. Peterson grading scale was used to indicate scar severity and nerve adherence as a macroscopic assessment (Table 1) (14). In histological assessment, 12-micrometer paraffin cross sections which encompass nerve, scar and surrounding muscles were stained by Masson trichrome and the surface area of scar to nerve was calculated as a ratio (15). The left hind limbs of four animals from each group which sustained no intervention, served as control group.

Two-way ANOVA was used for statistical analysis of functional assessment (TOA), followed by Bonferroni’s test as multiple comparison of means. One-sample T test was used to compare TOAs with the normal baseline. Correlations between TOA and Peterson, and TOA and scar index were analyzed by Spearman test. Measurements were considered significant when *P*<0.05.

## Results

**Figure 1:**
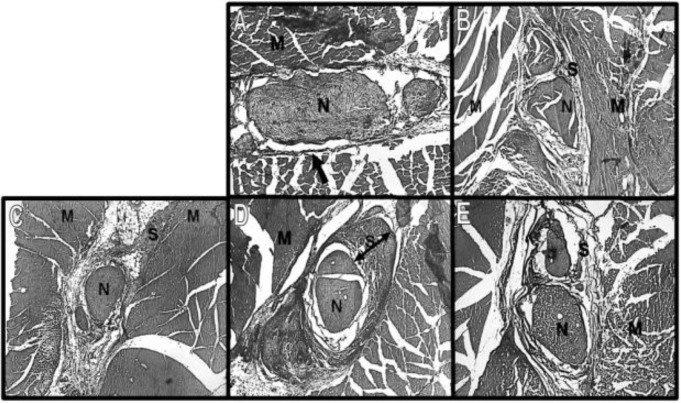
Photomicrograph of sciatic nerve (N) and surrounding scar (S) and muscles (M) in third week. A: control, B: laceration, C: crush, D: mince and E: burn. In the normal tissue, note the thin darkly stained collagen fibers of epineurium (arrow), which in the scarred tissue becomes a dense band around the nerve (two-head arrow). Masson trichrome staining. (Magnification A: ×100, B-E: ×50

Mean TOA of right hind limb which was subjected to scar inductions, showed no significant differences neither among the groups, nor between experimental groups and normal baseline for four weeks of follow up (*P*=0.78, Table 2). In macroscopic assessment, skin and muscle fascia were entirely closed in all animals during the study, while nerve adherence existed and showed variations among experimental groups (Table 3). Masson trichrome A thick layer of dark blue tissue was observed and measured with different intensities in experimental groups (data not shown) which histologically confirm formation of scar (Figure 1). Using Spearman test TOA had no correlation with either scar index or Peterson scores within the groups (*P*=0.083).

**Table 1:** Numerical grading scheme of peterson for gross evaluation of scar

Tissue	Grade	Definition
Skin and muscle fascia	1	Skin or muscle fascia entirely closed
	2	Skin or muscle fascia partially open
	3	Skin or muscle fascia completely open
Nerve adherence and nerve separability	1	No dissection or mild blunt dissection
	2	Some vigorous blunt dissection required
	3	Sharp dissection required

**Table 2:** Toe out angle of right hind limb. Values present mean ± SD. Normal toe out angle was measured 12.6 ± 4.1

Week	Laceration	Crush	Mince	Burn
1	12.5 ± 5.4	11.9 ± 3.2	13.1 ± 5.8	13.5 ± 1.5
2	9.9 ± 4.6	12.7 ± 3.5	10.5 ± 1	10.6 ± 4.5
3	13.9 ± 3	17.2 ± 2.3	10.2 ± 3.4	12.8 ± 6.5
4	10 ± 1.9	13.1 ± 0.1	9.9 ± 6.9	12.6 ± 10.3

## Discussion

This study aims to evaluate the translational capacity of four animal models of perineural scar formation in the sciatic nerve bed.Our results demonstrated no correlation between macroscopic or histological findings, and functional assessment (TOA). This suggests that function of the sciatic nerve is not affected by scar formation in spite of macroscopic and microscopic evidences which shows presence of intensive scar. 

Perineural scar formation is one of the major problems in peripheral nerve surgery by its potential hazards such as adhesion, tethering and compression of the nerve; all may lead to further functional deficit (10). Chronic nerve injury due to tethering and compression of the nerves is a central feature of clinical defects, a condition that would be ideal to achieve in animal models (16). Rodents, and especially rat, have been used in many scar formation models of preclinical research. This is mainly because of anatomic and physiologic similarities with human as well as ease of handling and being large enough for surgical interventions (17). Several studies have implemented scarring methods such as laceration (12), abrasion (9-11), crush (8) and burning of muscles (1, 8, 9). In our study these procedures were engaged as conventional physical ways for induction of scar. However, mincing of muscles as a new method was proposed by authors to maximize scar tissue and imitate tissue debris which usually exists after a traumatic injury in human. 

We used TOA to assess damage to the sciatic nerve which has correlation with sciatic functional index (SFI), a universally employed assessment for measuring sciatic nerve function in rat (13). TOA is advantageous in that it is less affected by technical difficulties in analysis of foot print due to flexion contractures and automutilation (13, 18). Although histological evaluation in this study describes the pattern and distribution of scar formation around the nerve after all interventions, based on functional results it appears that true modeling of nerve injury mechanisms is more than just induced morphological fibrosis. These shortcomings in the current models may root from differences in local biomechanical tissue environment between rat and human. 

One possible difference lays in the bone and soft tissue elements at the site of injury. For instance in rats, unlike clinical cases such as carpal and cubital tunnel syndromes, there is less chance of mechanical deformation through extrinsic forces imposed to sciatic nerve, perhaps due to its less muscular bulk and/or lack of enclosed bony tunnel in its path (4, 19). On the other hand, because of small anatomical size and more stable mechanical environment around the sciatic nerve of rat, adhesion of nerve to its surrounding, which limits its gliding, is less probable to cause injurious nerve tethering (20). 

**Table 3:** Scores of gross evaluation of scar in Peterson’s scale

Experimental groups	Skin and muscle fascia					Nerve adherence			
	Weeks					Weeks			
	1	2	3	4		1	2	3	4
Laceration	1	1	1	1		1	1	2	2
Crush	1	1	1	1		2	1	1	1
Mince	1	1	1	1		3	3	3	3
Burn	1	1	1	1		3	2	2	2
									

Regarding these differences, the base level of outcome of a therapeutic intervention may be overestimated when such animal models are implemented.

Taking to account the high healing capacity in rodents, it seems difficult to mimic extensive tissue damage around their peripheral nerve, like those caused by traumatic injuries in humans (4, 17, 21). While mincing model which proposed in this study did not reproduce all of the elements of an optimal model for scar formation, macroscopic and microscopic findings suggest a qualitatively intensive scar around the sciatic nerve.

## Conclusion

In conclusion, none of the animal models mentioned in this paper can reproduce all features of the human injury condition of scar formation around the nerve. More collaboration between basic and clinical scientists is needed to overcome the limitations of rat model and develop translational pathways to reach bench-to-bedside therapies for disabled patients.

## References

[1] Ohsumi H, Hirata H, Nagakura T, Tsujii M, Sugimoto T, Miyamoto K (2005). Enhancement of perineural repair and inhibition of nerve adhesion by viscose injectable pure alginate sol. Plast Reconstr Surg..

[2] Lundborg G, Dahlin LB (1992). The pathophysiology of nerve compression. Hand Clin.

[3] Saunders FW (1980). Scar prevention in peripheral nerve surgery. Can J Neurol Sci.

[4] Muschler GF, Raut VP, Patterson TE, Wenke JC, Hollinger JO (2010). The design and use of animal models for translational research in bone tissue engineering and regenerative medicine. Tissue Eng Part B Rev.

[5] Cheng DS, Rogers J, Wheeler A, Parker R, Teixeira L, Light RW (2000). The effects of intrapleural polyclonal anti-tumor necrosis factor alpha (TNF alpha) Fab fragments on pleurosdesis in rabbits. Lung.

[6] Bilaceroglu S, Guo Y, Hawthorne ML, Zhu Z, Stathopoulos GT, Lane KB (2005). Oral forms of tetracyclin and doxycyclin are effective in producing pleurodesis. Chest.

[7] Watcher BG, Leonetti JP, Lee JM, Wurster RD, Young MR (2002). Silver nitrate injury in the rat sciatic nerve: A model of facial nerve injury. Otolaryngol Head Neck Surg.

[8] Finsterbush A, Porat S, Rousso M, Ashur H (1982). Prevention of peripheral nerve entrapment following extensive soft tissue injury, using silicone cuffing; an experimental study. Clin orthop relat res.

[9] Gorgulu A, Uzal C, Doganay L, Imer M, Eliuz K, Cobanoglu S (2003). The effect of low-dose external beam radiation on external scarring after peripheral nerve surgery in rats. Neurosurgery.

[10] Ilbay K, Etus V, Yildiz K, Ilbay G, Ceylan S (2005). Topical application of mitomycin C prevents epineural scar formation in rats. Neurosurg Rev.

[11] Palatinsky EA, Maier KH, Touhalisky DK, Mock JL, Hingson MT, Coker GT (1997). ADCON-T/N Reduces in vivo perineural adhesions in rat sciatic nerve reoperation model. J Hand Surg Br.

[12] Sato K, Li Y, Foster W, Fakushima K, Badlani N, Adachi N (2003). Improvement of Muscle Healing Through Enhancement of Muscle Regeneration and Prevention of Fibrosis. Muscle Nerve.

[13] Varejo ASP, Cabrita AM, Geuna S, Melo-Pinto P, Filipe VM, Gramsbergen A (2003). Toe out angle; a functional index for the evaluation of sciatic nerve recovery in the rat model. Exp Neurol.

[14] Peterson J, Russel L, Andrus K, MacKinnon M, Silver J, Kilot M (1996). Reduction of external scarring by ADCON-T/N after surgical intervention. Neurosurgery.

[15] Ozgenel GY (2003). Effects of hyaluronic acid on peripheral nerve scarring and regeneration in rats. Microsurgery.

[16] Wu X, Gao Z, Song N, Chua C, Deng D, Cao Y (2007). Creating thick linear scar by inserting a gelatin sponge into rat excisional wounds. Wound Repair Regen.

[17] Dobkin BH (2007). Curiosity and care: translational research strategies for neural repair-mediated rehabilitation. Dev Neurobiol.

[18] Varejo AS, Melo-Pinto P, Meek MF, Filipe VM, Bulas-Cruz J (2004). Methods for the experimental functional assessment of rat sciatic nerve regeberation. Neurolog Res.

[19] Hunter JM (1991). Recurrent carpal tunnel syndrome, epineural fibrous fixation, and traction neuropathy. Hand Clin.

[20] McLellan DL, Swash M (1976). Longitudinal sliding of the median nerve during movements of the upper limb. J Neurol Neurosurg Psychiatry.

[21] Derwin KA, Baker AR, Lannotti JP, McCarron JA (2010). Preclinical models for transplanting regenerative medicine therapies for rotator cuff repair. Tissue Eng Part B Rev.

